# Therapy response assessment with quantitative PET: evaluation of a shortened acquisition protocol with dynamic PET/CT

**DOI:** 10.1186/1470-7330-14-S1-S1

**Published:** 2014-10-09

**Authors:** C Pfannenberg, SC Schüle, C Brendle, J Schwenck, K Nikolaou, C La Fougère, J Kupferschläger

**Affiliations:** 1 Department of Radiology, Eberhard-Karls-University Tuebingen, Germany

## Purpose

The results of SUV quantification for prediction of histopathological response in patients with oesophageal carcinoma show high variations with different accuracy. However, the routine use of a full dynamic PET is limited because of long acquisition times. We tested a shortened acquisition protocol for quantitative PET to overcome that limitation.

## Material and methods

13 patients with histopathologically proven oesophageal adenocarcinoma underwent a combined dynamic and static ^18^F-FDG PET/CT including CT tumour perfusion (Siemens, Biograph mCT). Dynamic PET (listmode) was acquired for 60 min resulting in 38 frames from 10 to 600 sec duration for the full dynamic dataset and 2 frames each with 600 sec duration (20-30 min and 50-60 min p.i.) for dual time point PET (DTP). We evaluated the metabolic rate Ki using different models: 2-compartment irreversible model (Fit), Patlak plot and DTP (van den Hoff et al). The CT tumour perfusion protocol included the parameters blood flow, blood volume and permeability.

## Results

The metabolic rate Ki could be reliably reproduced independent of the analytical model; we observed only slight variations of Ki with respect to the analytical model: -4,9% (Patlak vs.Fit), -10% (DTP vs.Fit) and -5,1% (DTP vs.Patlak). A linear regression revealed a strong correlation of the Ki values: R² = 0,996 (Patlak vs. Fit), R² = 0,968 (DTP vs.Fit) and R² = 0,985 (Patlak vs. DTP).

## Conclusion

The shortened dynamic acquisition protocol of DTP-PET is a reliable method for the determination of the metabolic rate Ki and can substitute a full dynamic scan for improved quantitative assessment of therapy response.

